# A qualitative study of the barriers and facilitators to adopting a sustainable diet among adolescents in Ireland

**DOI:** 10.1371/journal.pone.0361509

**Published:** 2026-10-27

**Authors:** Connor Dupuits, Amanda McCloat, Elaine Mooney

**Affiliations:** Department of Home Economics, National Centre of Excellence for Home Economics, Atlantic Technological University, St. Angelas, Sligo, Ireland; International Medical University, MALAYSIA

## Abstract

An increasing body of evidence indicates that systemic changes to the food system are necessary to mitigate ongoing environmental degradation. The adoption of sustainable dietary practices is an actionable individual-level strategy that may benefit both human health and environmental sustainability. This paper aimed to explore the barriers and facilitators to adopting a sustainable diet among adolescents in Ireland. The data from this research seeks to clarify the steps needed to support this dietary transition. Data were collected from four sites, involving 48 participants aged 15–17 years in seven semi-structured focus groups. Non-probability convenience sampling was employed. Transcripts were audio-recorded, transcribed, and inductively coded to identify barriers and facilitators, which were then deductively mapped onto the Theoretical Domains Framework and presented on the Capability, Opportunity, Motivation – Behaviour model. Barriers (*n* = 33) were more frequent than facilitators (*n* = 8) in the adoption of a sustainable diet. Prominent barriers to adopting a sustainable diet included a lack of knowledge about the characteristics of a sustainable diet, a current diet that does not align with a sustainable diet, a lack of practical skills for preparing a sustainable diet, unaccommodating school canteens, and cultural influences such as a strong attachment to meat. Facilitators to the adoption of a sustainable diet were the view that this diet promotes human health, and the acknowledgement of the changes required to make their diet more sustainable. However, although participants could identify changes to make their diets more sustainable, most were unwilling to transition at present. This research is important because it provides insights into the design of future interventions using the Behaviour Change Wheel intervention framework to facilitate behaviour change to support adolescents’ transition to a sustainable diet.

## Introduction

The urgency to shift to a sustainable diet is growing and is essential to nourish a growing population without harming the planet [1–4]. The Food and Agriculture Organization (FAO) and the World Health Organization (WHO) define a sustainable diet as *“dietary patterns that promote all dimensions of individuals’ health and wellbeing; have low environmental pressure and impact; are accessible, affordable, safe and equitable; and are culturally acceptable”* (p. 9) [2]. Furthermore, FAO and WHO [2] emphasise that the objective is to attain optimal health for both current and future generations while safeguarding the planet’s health. Consequently, significant changes are necessary at the policy and individual levels.

Despite growing evidence of the unsustainability of the current food system and consumption patterns, reviews have identified barriers to sustainable diets, including consumers’ limited knowledge of what constitutes a sustainable diet [5–7]. From an international perspective, Alnasser and Musallat [8] found that fewer than 40% of individuals knew the term ‘food sustainability.’ Further studies all report that consumers displayed low awareness of sustainable diets [9–13].

Similarly, among the few studies conducted in Ireland, awareness of a sustainable diet also appears low [14, 15]. Reviews have also reported barriers to consumers’ intention to adopt a sustainable diet, with participants citing factors that hinder their likelihood of adopting such a diet [5–7,16]. For instance, a systematic review [17] identified environmental and nutrition education and awareness, financial incentives, and community engagement as enablers towards the adoption of a sustainable diet. However, this same review reported barriers to a sustainable diet, such as high cost, cultural resistance to change, and limited access to nutritious options [17].

Notably, research conducted on adolescents is limited. However, of the available research, a review exploring interventions and perceptions amongst adolescents reported that adolescents’ knowledge of sustainable diets is poor [18]. Further, current behaviour and willingness to adopt a sustainable diet appear even lower, and adoption remains a current challenge.

Given the sustainable diets’ emphasis on large quantities of fruit and vegetables, a sustainable diet already appears challenging for adolescents. For instance, internationally, Rakić et al. [19] found that only 38% of adolescents consume fruit and vegetables daily. In Ireland, adolescents display similarly low intake, with fewer than three servings per day [20]. Consequently, there is a misalignment with The Eat Lancet report [4], who recommend that fruit and vegetables should constitute half of total food intake, and the average adolescent's diet. This misalignment in adolescents’ current diet in Ireland extends beyond fruit and vegetable intake. A study by Poore and Nemecek [21] found that animal products, particularly beef, are the most environmentally damaging in terms of greenhouse gas (GHG) emissions and land use compared to plant-based proteins. Again, a discrepancy emerges between the diets recommended by Willett et al. [1] and Rockström et al. [4] and the diet of Irish adolescents, with data indicating that adolescents overconsume meat and underconsume plant-based proteins [20].

Thus, dietary modifications are vital for improved human and planetary health. However, concerning willingness to adopt a sustainable diet, findings reported by Lourenco et al. [12] and Varela et al. [22] demonstrated high levels of resistance to adopting a sustainable diet and showed no intention of transitioning to it. Thus, there seems to be a scenario where both knowledge and intentions to adopt a sustainable diet appear low.

Therefore, an intervention may facilitate the transition towards a sustainable diet. The Behaviour Change Wheel (BCW) is a framework for developing and adapting behaviour change interventions. It was developed from 19 behaviour change frameworks [23]. As a result, BCW identified common features across these frameworks to build a behavioural model applicable across various settings [24]. The BCW centres on the COM-B (Capability, Opportunity, Motivation–Behaviour) model, which captures human behaviour [23]. The COM-B model can be expanded using the Theoretical Domains Framework (TDF), with the 14 TDF domains mapped to the COM-B components [25]. See Fig 1 for a diagram depicting COM-B and TDF.

**Fig 1:**
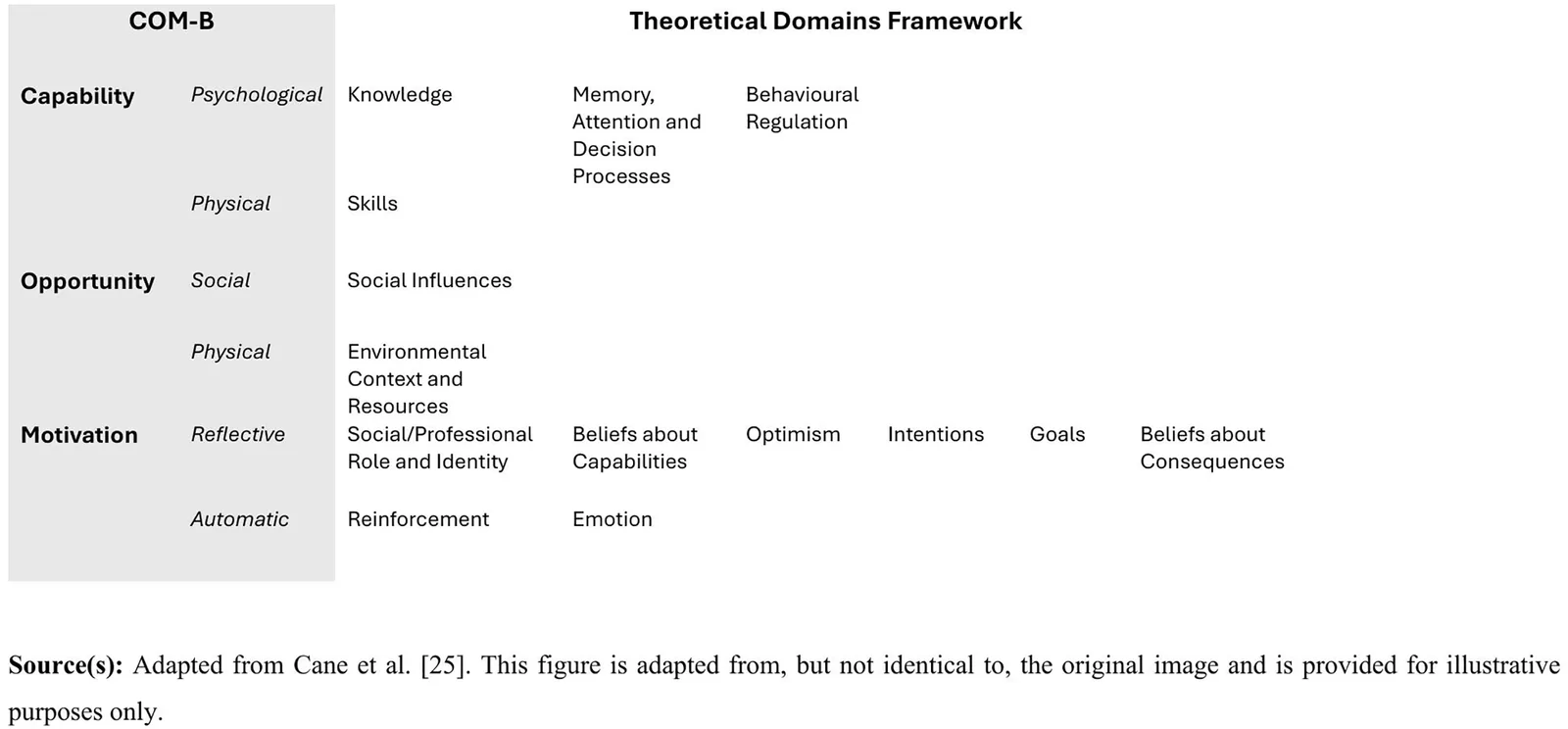
Theoretical Domains Framework mapped onto the COM-B model.

Each of the three COM-B components links to specific domains of the TDF. Capability refers to an individual's physical and psychological capacity to perform a behaviour. Physical capability includes skills, while psychological capability encompasses knowledge, behavioural regulation, memory, and decision-making processes. Both forms of capability influence whether a person can engage in a particular behaviour effectively [25]. Opportunity refers to physical and social external factors that enable or constrain behaviour. Physical opportunity includes resources, time, environmental conditions, and access to facilities, whereas social opportunity involves cultural norms, social influences, support from others, and interpersonal relationships [25]. Motivation encompasses the mental processes that direct and promote behaviour. Reflective motivation involves conscious planning, evaluation, intentions, and beliefs, while automatic motivation includes emotions, reinforcement, habits, and routines that operate with less conscious awareness. Together, these processes influence the likelihood that behaviour will be initiated and maintained [26].

The TDF was selected as it provides a comprehensive, theory-informed approach to understanding behaviours that influence complex health behaviours. By encapsulating constructs from multiple behaviour change theories into 14 domains, the TDF enables the identification of barriers and facilitators towards the adoption of a sustainable diet [26]. The COM-B model complements the TDF by organising these determinants into three interacting components, together influencing behaviour [25]. Applying the TDF and COM-B is particularly appropriate in the context of sustainable dietary behaviours, as food choice is shaped by a myriad of factors. Furthermore, adolescence represents a vital developmental period where lifelong dietary habits are established, making the use of theory-informed behavioural frameworks valuable for understanding the factors influencing the adoption of a sustainable diet and for informing the creation of targeted interventions. Additionally, although studies have examined consumer knowledge and willingness to adopt a sustainable diet, there is a dearth of research in Ireland and a limited focus on adolescence [7]. Fundamentally, this study aims to explore the barriers and facilitators to adopting a sustainable diet among adolescents in Ireland.

## Materials and methods

### Ethical approval

Ethical approval was granted by the Atlantic Technological University (ATU) Research Ethics Committee on 3 March 2025 (Reference number: 2025007). Written informed consent was obtained from parents or legal guardians, and written assent was obtained from all adolescent participants prior to data collection. Participants were informed of the voluntary nature of the study and their right to withdraw at any time without consequence. All data were anonymised to ensure confidentiality and stored securely in accordance with data protection regulations.

### Participant recruitment and sampling methods

The included participants were between the ages of 15 and 17. Therefore, as participants were children, the researchers followed the Department of Children and Youth Affairs (DCYA) guidance on developing ethical research projects involving children throughout the study [27].

A non-probability convenience sampling approach was used, with schools selected based on their accessibility and willingness to participate. This sampling strategy was appropriate for the exploratory qualitative design, prioritising the collection of rich, context-specific data over statistical representativeness. The recruitment process began on March 5^th^, 2025, and ended on May 5^th^ 2025. Participants were eligible if they were enrolled in an Irish secondary school to ensure they shared a common educational and environmental context relevant to the study aim. Participants were also eligible if they were in their senior cycle, as they are more likely to possess greater cognitive capacity to discuss sustainability concepts and have greater independence in making food choices than younger adolescents. Participants were therefore excluded if they were not enrolled in an Irish secondary school or in junior cycle years.

Principals were contacted and asked for permission for their students at their schools to be involved in the study. Agreeing principals were then asked to appoint a gatekeeper to select participants who met the inclusion criteria. Written parental consent, written student assent forms, and participant information sheets were provided to the gatekeeper to administer to nominated participants. The gatekeeper also handled the collection of the completed assent and consent forms. Upon the researchers’ arrival at the participating schools, all signed consent and assent forms were collected before beginning the focus groups.

### Research method and tool

Semi-structured focus groups with open-ended questions were selected as the most appropriate method for this age group. This is because children tend to feel more at ease sharing opinions when discussions involve peers [28]. Furthermore, Gibson's [29] guidelines for conducting focus groups with children were utilised to develop the research tool. The research tool consisted of questions that were developed in collaboration with the TDF.

A total of 12 questions were asked, which covered all 14 TDF components. The first question focused on their existing knowledge of a sustainable diet: “What do you know about the term ‘sustainable diet’?” This initial question was asked before the participants received any theoretical input on a sustainable diet. Subsequently, the researchers introduced the concept and presented a visual representation of a sustainable diet before proceeding (S1 File). The next 11 questions focused on their opinion of the outlined sustainable diet, their perceptions of a sustainable diet, and their perceived barriers and facilitators to adopting a sustainable diet. A combination of note-taking and audio recording collected data. Each focus group was expected to take an hour. The visual aid used was adapted from Willett et al. [1]. The questions asked throughout the focus group are presented in Table 1. A more detailed version of the focus group schedule is outlined in S1 File.

**Table 1:** Focus group questions asked to participants in accordance with the TDF.

Questions asked throughout the focus groups
What do you know about the term ‘sustainable diet’? • Prompt: What do you think are the features/characteristics of a ‘sustainable diet’ • Prompt: How do you think this diet compares to a healthy diet?
*Teaching of a sustainable diet*
What is your opinion of a sustainable diet? • Prompt: What is your initial reaction to a sustainable diet?
How do you think a sustainable diet could impact your health? • Prompt: Not applicable
How do you think red meat production affects the environment? • Prompt: Do you think these effects are mostly positive or negative or neutral? Explain
What are the factors that affect the foods you eat? • Prompt: Do you set any personal goals regarding your dietary choices?
What are some challenges you think people your age may face if they want to eat a more sustainable diet? • Prompt: Not applicable
How do you think your current diet/food choices might affect the environment? • Prompt: How do your diet/food choices compare to the sustainable diet discussed?
What is the single biggest thing you could do to make your current food choices more sustainable? • Prompt: Do you find it easy to remember or plan to include sustainable foods in your meals? What helps or hinders you?
If you had to adopt a sustainable diet, how would you feel about doing so? • Prompt: What would be your initial reaction if you had to adopt a sustainable diet? • Prompt: Can you see eating sustainably as part of who you are, or as part of your identity?
Is it realistic for you to follow? Explain • Prompt: How confident are you that you or people your age could successfully adopt a sustainable diet?
What would be the biggest challenge, if any, for you if you were to adopt a sustainable diet? • Prompt: Not applicable
Are there rewards or positive outcomes that would encourage you to eat a sustainable diet? • Prompt: Not applicable

The focus groups were pilot tested in March 2025 to assess the adequacy of the focus group questions and confirm their relevance [30]. Two pilots were conducted, consisting of a group of six and a group of seven students who met the inclusion criteria. These participants were recruited in the same manner as the recruitment process for the included focus groups; one school was used for the pilot. During both feedback sessions with the participants, the primary concern was the wording of some of the questions, which were pitched too high for some participants. An example was the change from ‘What is the primary adaptation you could make to your food choices to make them more sustainable?’ to ‘What is the single biggest thing you could do to make your current food choices more sustainable?’ as some experienced confusion regarding the term ‘primary adaptation.’ Another suggestion was the need for examples of each food group that belongs to the components of a sustainable diet. Both concerns were rectified in the final focus group schedule.

### Data analysis

An interpretivist paradigm grounded this research [31]. This means that the researchers explored how adolescents interpret sustainable eating and how they use their understanding to determine whether they would adopt a sustainable diet. Seven in-person focus groups were conducted and audio-recorded on Microsoft Teams, and the recordings were transcribed verbatim. All transcripts were uploaded to NVivo 14. To identify barriers and facilitators, the data were initially coded inductively, and each code was then deductively mapped onto the codes of the TDF and presented on the COM-B model. The definitions of the TDF domains proposed by Cane et al. [25] were considered. Data saturation was reached when no new codes emerged during the seventh focus group. Further information on the data coding and corresponding quotations is provided in S2 File. The Standards for Reporting Qualitative Research (SRQR) reporting checklist was used when editing the final manuscript [32].

### Reflexivity

The lead researcher (principal investigator) is a lecturer in home economics and education, which informed the study's design and interpretation and ensured attention to participants’ perspectives throughout the research process. The lead researcher facilitated all focus groups, with a research assistant documenting field notes. The researchers had no prior relationship with participants and were introduced solely as members of the research team to minimise potential influence on participants’ responses. To enhance the trustworthiness of the findings, data analysis was conducted collaboratively by three researchers. Regular discussions throughout the analytical process helped refine interpretations and enhance the credibility of the findings.

## Results

### Participants

A total of seven focus groups involving 48 participants aged 15–17 were conducted in schools across four sites from April 1, 2025, to May 16, 2025. All participants were under 18 years old, with 16-year-olds forming 60% of the group (*n* = 29). Also, 71% of participants attended town schools (*n* = 34), while the remaining attended a school in Dublin (*n* = 14). With respect to gender, females comprised the majority at 69% (*n* = 33). Additionally, only one participant practised vegetarianism. An outline of participant details is shown in Table 2.

**Table 2:** Participant Characteristics.

Participant Characteristics	Total N = 48	FG1 *n* = 9	FG2 *n* = 5	FG3 *n* = 6	FG4 *n* = 6	FG5 *n* = 8	FG6 *n* = 7	FG7 *n* = 7
Jurisdiction, *n*								
City	14	9	5	0	0	0	0	0
Town	34	0	0	6	6	8	7	7
Gender, *n*								
Male	15	0	0	0	2	4	5	4
Female	33	9	5	6	4	4	2	3
Age, *n*								
15	2	0	0	1	0	0	0	1
16	29	0	5	1	2	8	7	6
17	17	9	0	4	4	0	0	0
Vegan/Vegetarian, *n*								
Yes	1	0	0	0	0	1	0	0
No	47	9	5	6	6	7	7	7

A total of 44 barriers and facilitators were coded from the data. Barriers (*n* = 33) were more prominent than facilitators (*n* = 8). Three codes were deemed either a barrier or a facilitator. For example, the code “Influence of social media on food choices” could be interpreted as either a barrier or a facilitator to the adoption of a sustainable diet. The coding of the barriers and facilitators to the TDF of the COM-B model is depicted in Table 3.

**Table 3:** Coding of the barriers and facilitators to the TDF of the COM-B model.

COM-B Component	TDF Domain	Code	Barrier (B) or Facilitator (F)
Capability (Psychological)	Knowledge	Unfamiliarity with plant sources of protein	B
		Unawareness of the characteristics of a sustainable diet	B
		Unsure of how red meat production affects the environment	B
		Awareness that a sustainable diet keeps resources for future generations	F
		Understanding that a sustainable diet does not damage the environment	F
	Behavioural Regulation	Current diet does not align with a sustainable diet	B
		Acknowledgement of the changes required to make their diet more sustainable	F
Capability (Physical)	Skills	Lack of practical skills to prepare sustainable meals	B
Opportunity (Social)	Social Influences	Parents buying and preparing food	B
		Peer pressure shaping food choices	B
		Socially unacceptable	B
		Social norm clashes	B
		Influence of social media on food choice	B/F
		Socially acceptable	F
Opportunity (Physical)	Environmental Context & Resources	Lack of access to sustainable foods	B
		The school canteen has limited options	B
		Convenience as a factor affecting food choices	B
		Inconvenience of a sustainable diet	B
		Stage of life clashing with a sustainable diet	B
		Cost shaping food choices	B/F
Motivation (Reflective)	Beliefs about Consequences	The perception that a sustainable diet would not be satiating	B
		Perceiving a sustainable diet as expensive	B
		The amount of dairy/meat/starch is too small	B
		A lot of ‘harmful’ oils	B
		Concerns surrounding the generalisability of a sustainable diet	B
		Sustainable diet adoption is adversely impacting the Irish economy	B
		Belief that red meat production has a positive environmental impact	B
		Belief that red meat production has a neutral environmental impact	B
		Transportation of vegetables is worse than that of local meat	B
		The healthiness of food determines food choices	F
		Belief that red meat production has a negative environmental impact	F
		Viewing a sustainable diet as positive for human health	F
	Social/Professional Role & Identity	Cultural influence on diet	B
		Attachment to meat and dairy	B
		Attachment to starch	B
	Beliefs of Capabilities	Struggle to reduce meat and dairy	B
		Lack of confidence to change diet	B
		Diet is too dissimilar to make a change	B
		Believing change is possible	F
	Intentions	Unwillingness to adopt a sustainable diet	B
		Change would take time	B/F
Motivation (Automatic)	Emotion	Dislike of vegetables	B
		Negative reaction to following a sustainable diet	B
		Shocked at the number of plant-sourced proteins in a sustainable diet	B

### Capability

Concerning psychological capability – knowledge and behavioural regulation, were reported to act as both barriers and facilitators to the adoption of a sustainable diet. On the other hand, physical capability – skills, was reported as a main barrier to sustainable diet adoption.

#### TDF-knowledge.

While reporting their understanding of a sustainable diet, knowledge varied, acting as both a potential barrier and a facilitator. For instance, only one participant in each focus group associated a sustainable diet with environmental protection, with the rest unaware of the term. The few who understood a sustainable diet described it as eating locally, using less packaging, minimising food waste, ensuring resources for future generations, and eating seasonal foods.

*“A diet you can eat that can keep you healthy, as well as not negatively impact the environment.*” (FG1, P6)

Conversely, most participants were unable to describe the characteristics of a sustainable diet because many had never encountered the term. Some associated a sustainable diet with being ‘sustained’ or simply with being maintainable, without considering the environment. Some also admitted they could not identify plant-based proteins, like tofu, and a few were unaware that protein can be found in plant sources.

*“I did not know that beans had protein in them, I won’t lie.”* (FG7, P3)

Notably, no participants had knowledge of reducing meat consumption as part of a sustainable diet, nor did they mention environmental concerns as a factor influencing food choices. Their knowledge of the environmental impact of cattle production was controversial, with responses ranging from positive to negative. Although participants recognised the environmentally damaging effects of red meat production, many were uncertain and could not provide specific examples.

*“I don't know too much about it, but I do know that there's a whole thing going on, the gases that cows produce, like it's damaging the environment. So, I think that would be like an environmental reason why people would try to cut down on their consumption of beef. But I don't know so much about it.*” (FG3, P6)

#### TDF-behavioural regulation.

An evident barrier was that the participant's diet does not align with a sustainable diet. To assess differences between participants’ diets and the visual representation of a sustainable diet outlined by the EAT-Lancet Commission, participants were asked to identify any discrepancies between their current eating habits and those recommended by the EAT-Lancet Commission. Interestingly, only the participant following a vegetarian diet reported that their diet was similar to the proposed sustainable diet, whereas the vast majority identified at least one, and often many, mismatches. The most frequent discrepancies included high consumption of animal products, excessive intake of sugar and starchy foods, insufficient consumption of vegetables and unsaturated plant oils, and a general lack of plant-based protein.

*“My animal protein would probably be like a quarter of a plate.”* (FG3, P4)

However, a potential facilitator was the participants’ acknowledgement of the changes required to make their diet more sustainable. When asked to suggest potential changes to make their diet more sustainable, participants recognised the need to reduce consumption of foods they consumed excessively, such as meat and dairy, and to increase consumption of foods they consumed less often, such as pulses, vegetables, and wholegrains.

*“More plant-sourced proteins as well. I could throw a few beans into something.”* (FG6, P5)

#### TDF- skills.

Another apparent barrier to the adoption of a sustainable diet included a lack of culinary skills in using plant-based proteins, with many reporting that they cannot effectively replace animal-based proteins with plant sources.

*“A lot of parents only eat animal sources of protein; let’s say they don't know how to cook tofu, and then they can't use it.”* (FG4, P6)

### Opportunity

Social opportunity – social influences, and physical opportunity – environmental context and resources, were reported as barriers. The relationships between factors affecting food choices and barriers to adopting a sustainable diet were similar. The main social influence barriers were parents’ role as primary food providers at home, peer pressure, social acceptance, and social norms. The dominant environmental context and resource barriers were a lack of access to sustainable food options, unaccommodating school canteens, convenience, cost, and their current stage of life.

#### TDF-Social influences.

Concerning social influences, barriers significantly outweighed facilitators towards the adoption of a sustainable diet.

Parental control over household food purchases was a notable barrier identified.

*“You can only really eat what your parents are buying and making for you, so if you want to eat like this [sustainably], and your parents are like, I am making this dinner, and you can either eat it or not eat it.”* (FG4, P2)

Regarding social acceptance, most participants considered this diet socially unacceptable among their peers and reported feeling embarrassed about following it.

*“You would get slagged if you've got a big bundle of salad, you would get called a ‘rabbit’ or something.” (*FG5, P8)

Furthermore, participants also noted clashes in their social norms and a discrepancy between what they had been told and the proportions of each food in a sustainable diet.

*“You need meat to grow, especially when you're young, so my mam always says.”* (FG2, P5)

However, some participants expressed how expectations surrounding social acceptance would not be the ultimate barrier towards the adoption of a sustainable diet.

*“I think if you're surrounded by the right friend group, nobody would judge for what you decide to eat, you know.”* (FG3, P2)

Another potential facilitator could be the potential benefit that social media may have in promoting and supporting sustainable diets. However, this may also act as a potential barrier, as the converse is also true.

*“If you're seeing more unhealthy things, you might want to eat less healthy. But if you see more people like influencers, eating healthy might influence you to eat healthier food.”* (FG3, P6)

#### TDF-environmental context and resources.

Inconvenience, inaccessibility, and the lack of sustainable options at the school canteen were also significant barriers to adopting a sustainable diet.

*“There is not really a very healthy option [at school], as they don't do salads. They don't like things with vegetables in them; it's just animal protein they use.”* (FG1, P4)

*“A sustainable diet is less convenient because of its preparation; you have to peel most vegetables, wait for them to boil, and chop them. Whereas with meat, you can just put it in the oven or the pan.”* (FG3, P2)

*“Convenience, like. Say if you're out in town after school or something, a takeaway is handy instead of going into the shop and buying vegetables. You have to cut them up and make stuff out of them.”* (FG6, P6)

Additionally, although largely uninterested in adopting a sustainable diet at their current stage of life, a few participants noted that if they were older and responsible for purchasing their own food, it would be easier for them to follow one.

*“I think it'd be more realistic if I were older. If I like, live by myself or something. Or if I were making dinners for myself and I was on like, my schedule, it would be a lot easier.”* (FG2, P3)

### Motivation

Reflective motivation – beliefs about consequences, was reported to contain both barriers and facilitators, while social/professional role and identity, beliefs about capabilities, and intentions, were reported as barriers towards sustainable diet adoption. Likewise, automatic motivation – emotion, was a reported barrier.

#### TDF-beliefs about consequences.

A facilitator is that overall, participants believed that red meat production has harmful environmental effects. However, although few, some perceived red meat production as having a neutral or even positive environmental impact by helping to manage the overpopulation of cattle, with one participant denying that red meat production has adverse ecological effects.

*“They probably balance each other out, kind of, because they both can be negative. Ireland is known for beef and stuff. And then if you're bringing, like, avocados, from God knows whatever, you know, the travel costs the environment a lot to bring them over.”* (FG6, P7)

After the explanation and visual depiction of a sustainable diet, some expressed concerns about the absence of dairy and animal-sourced protein. Most regarded dairy as an essential source of calcium and animal-sourced protein as a primary source of protein and iron.

*“I just always thought that people were encouraged to eat meat because it's still good for you and has so much protein in it.”* (FG2, P3)

Additionally, some participants showed concerns about the adequacy of a sustainable diet, specifically whether it addresses dietary needs associated with pregnancy, heart disease, the life cycle, and osteoporosis.

*“Like children are obviously growing very fast, I don't think that'll be enough protein for a child, or like a pregnant woman who would need an awful lot more protein.”* (FG3, P2)

Additionally, the participants’ view that a sustainable diet would not be satiating also posed as a barrier to the adoption of a sustainable diet.

*“I think you would be a lot hungrier because I suppose the majority of my energy comes from starchy vegetables and stuff like that, and whole grains. But I just feel like I'd be so hungry if half my plate were vegetables. I’d feel like I'd have no energy.”* (FG3, P1)

Likewise, the perceived cost of a sustainable diet also deterred many participants from adopting a sustainable diet.

*“Just how expensive vegetables and fruit would be.”* (FG5, P4)

Participants also questioned the abundance of ‘unhealthy oils’ and the need to limit their consumption in the diet, as they believe these to be harmful. Confusion and disagreement arose among participants regarding other aspects of a sustainable diet, such as pricing, with some viewing it as costly and others as affordable.

*“The vegetables and fruit would probably be cheaper than what people are buying at the moment, and people would be saving money.”* (FG6, P2)

Another concern raised by participants was the potential impact on the Irish economy if the Irish population significantly reduced its consumption of meat and dairy products. Concerns were also voiced from an Irish export perspective regarding a potential decline in global demand for meat and dairy.

*“We transport a lot of meat, which means we get a lot of money, so part of our income comes from meat, so it has its positives as well. In Ireland, it's a large proportion of people's income.”* (FG4, P5)

Some also viewed this approach as counterintuitive and noted that Ireland’s climate is unsuitable for year-round cultivation of certain fruits and vegetables, which would likely increase imports if a sustainable diet were adopted. Furthermore, some argue that buying locally produced meat has a smaller environmental impact than importing plant-based foods.

*“If you grow fruit and veg, you can’t grow a lot of them in Ireland, and if you have to get them from other countries, that would be worse for flying them over or shipping, and that would be worse for the environment, I think.*” (FG6, P1)

A potential facilitator is that although many participants were sceptical that a sustainable diet could meet diverse dietary needs and considered it impractical for most, the vast majority stated how the health of a food determines their food choices and how they were drawn to the perceived health benefits they believed they would gain from adopting such a diet, as they were more motivated by human health than by environmental concerns.

*“It'd probably be better for your skin, for your teeth, for your hair, for your eye health, and probably be better for your gut too … it's also better for maintaining your weight, for losing weight.”* (FG1, P8)

#### TDF-social/professional role and identity.

A significant barrier participants reported towards the adoption of a sustainable diet was that it appeared to conflict with their cultural background and identity.

*“I think it probably would be [easy to adopt] if you’ve grown up with it, but because we have not grown up with it, it is not our comfort food. It's not like the cultural food that we've been eating.”* (FG1, P3)

Similarly, their diets have always been influenced by attachment to animal-sourced protein, dairy products, and starchy foods.

*“That could be very difficult because people are so used to eating what they’re eating, like especially animal-sourced protein, like meals revolve around that, like pick a meat out fridge and then go ok, I'm going to make something with this meat and then revolve the rest of my dinner around that.”* (FG4, P1)

#### TDF-beliefs about capabilities.

An additional barrier was a reported lack of confidence among participants in adopting a sustainable diet.

*“It sounds silly, but nervousness, like if you're changing up your entire diet and menu plan, I think it can be a bit daunting because if you're trying to do things you haven't tried before, it could be a waste of money if you buy certain foods and you don't like them.”* (FG4, P6)

Another finding was that current diets are far from recommendations for a sustainable diet, making it difficult to follow, especially since meat and dairy are viewed as too difficult to reduce.

*“I think the majority of people our age will at least eat meat with like two of their meals, like I could eat meat with all three of my meals. I could eat meat with my breakfast, my dinner, and my supper, so it'd be very difficult to cut down on that when it's a big focus on the majority of my meals, like.”* (FG3, P5)

Although the vast majority of participants admitted that they would find it difficult to align their diet to a sustainable diet, one participant indicated that using tofu as a meat substitute to reduce meat consumption would be easy.

*“I feel like that wouldn't be terribly hard, though, because you can use tofu as a replacement for a lot of different types of meat, and then lentils. You can't replace them the same way as you could replace chicken or something; you can add it into sauces.”* (FG1, P9)

#### TDF-intentions.

The most prominent barrier was the notion that the majority still did not consider a sustainable diet feasible for them and therefore had no plans to adopt one.

*“Like, obviously, there's always a select few that are different. But I think the majority have no intention of following that [sustainable diet].”* (FG3, P4)

Conversely, a few undecided participants cautiously indicated that they might adopt a sustainable diet if they gradually transitioned to it.

*“I would have to change slowly into it because it's just not sustainable if I were to hop straight into the diet; it's just not what I'm used to.”* (FG7, P2)

#### TDF-emotion.

Concerning participants’ feasibility and readiness to adopt a sustainable diet, willingness to adopt a sustainable diet was rare, some were undecided, and the vast majority were strongly opposed to adopting a sustainable diet.

*“I’d hate it if I had to eat like that.”* (FG2, P5)

Regarding the composition of a sustainable diet, the inclusion of plant-based protein and large quantities of fruit and vegetables also elicited irritation among some participants.

*“I am shocked there is that much veg. Like I knew there'd be a lot, but I didn't think they'd be half your diet.”* (FG2, P4)

## Discussion

The study aimed to explore the barriers and facilitators to adopting a sustainable diet among adolescents in Ireland**.** Overall, the main findings of this study highlighted that barriers (*n* = 33), such as lack of knowledge of the characteristics of a sustainable diet, participants’ current diet not aligning with a sustainable diet, lack of practical skills regarding the preparation of a sustainable diet, lack of sustainable food options in school canteens, and cultural influences such as a strong attachment to meat and dairy, were more prominent that facilitators (*n* = 8) to adopting a sustainable diet. Furthermore, misconceptions about sustainable diets were common, with many participants believing that large amounts of meat and dairy are essential, or that plant-based diets do not provide enough protein. In addition, after receiving theoretical input about sustainable diets, many participants still expressed concerns, such as the limited amount of animal sources. However, although some facilitators were identified, such as the view that this diet promotes human health, and the acknowledgement of the changes required to make their diet more sustainable, they were less influential. Moreover, although most participants were able to recognise changes that they could make to improve the sustainability of their diets, most were not willing to make these changes at present.

Beginning with capability, psychological and physical capability served as barriers and facilitators towards the adoption of a sustainable diet. Exploring psychological capability – knowledge, confusion, and a lack of knowledge about sustainable diets were evident throughout the data. Although some participants associated a sustainable diet with balancing human and planetary health, none mentioned reducing meat consumption; instead, they focused on aspects such as eating locally and purchasing food in season. Likewise, Hazley et al. [15] found that Irish consumers overestimate the importance of eating organic and locally and underestimate the environmental impact of consuming red meat.

However, a further barrier was that most participants were unaware of the key characteristics of a sustainable diet. Likewise, numerous studies conducted on adults have reported low consumer awareness of sustainable diets [9 - 13]. Furthermore, Kirbis et al. [33] observed that nearly 74% of participants had a poor understanding of the environmental aspects of diet. It has also been reported that adolescents struggle to understand what constitutes a sustainable diet [18, 34]. Additionally, when asked how they believe red meat production affects the environment, a facilitator was that many cited adverse effects; however, their responses were superficial and lacked depth. Many could only state that red meat production harms the environment but were unable to give specific examples. Additionally, Polleau and Biermann [35] and Hazley et al. [15] found low levels of awareness regarding the environmental impact of meat in adult diets. Likewise, Havermans et al. [36] reported that adolescents have limited knowledge of a plant-based diet. Therefore, as summarised by Haghighian Roudsari et al. [37], increased awareness is necessary regarding what constitutes a sustainable diet. However, regarding psychological capability – behavioural regulation, once provided with a definition, although most participants remained reluctant to adopt a sustainable diet, they were capable of discussing the changes they would need to make if required to do so.

Interestingly, another barrier was that none of the participants cited environmental factors as influencing their food choices. Likewise, young adolescents did not report sustainability as a factor when making food choices [38]. Gelinder et al. [39] also reported that adolescents consider sustainable choices but ultimately select taste as the determining factor. Conversely, Eckert et al. [13] found health and environmental reasons to be the primary motivators for adopting a sustainable diet among young adults. Additionally, Ruzgys and Pickering [40] reported that environmental factors were important determinants of young adult participants’ dietary choices. Therefore, people usually overlook environmental factors when making food choices, highlighting a gap between their knowledge and behaviour.

Physical capability – skill, however, was reported as another barrier, with most admitting to lacking the skill and confidence to make these changes. This finding was similar to that of Ronto et al. [41], whose participants highlighted the need to reduce meat intake and increase consumption of plant-based foods. The ability to recognise and acknowledge discrepancies is a step toward a sustainable diet. However, physical skills as a barrier were apparent when participants were exposed to the visuals of a sustainable diet, such as the general lack of awareness and poor culinary skills related to preparing and cooking plant-based proteins, which are common barriers across many studies [12,13,22,36,41–43].

Exploring opportunity, both social and physical opportunity were largely reported as major barriers. The dominant factors influencing participants’ food choices are parental purchasing, social acceptance, convenience, food availability, cost, cultural influences, taste, social media, and school canteen options. These seem to function more as a sustainable diet. Similarly, a study on Irish teenagers identified sensory appeal, cost, availability, and health as key factors influencing food choices [44].

Regarding convenience, participants found preparing food for sustainable eating burdensome and time-consuming. Adolescents have also reported time as a potential barrier to ethical eating [45]. This has also been recognised by Mann et al. [9] as a potential barrier to adopting a sustainable diet. Participants also noted that the lack of healthy, sustainable food options is a barrier. Interestingly, cost was not considered a potential barrier to adopting a sustainable diet in this study. This contrasts with many other studies conducted on adults that do identify cost as a barrier [8,9,11,13,36,40,46,47]. Thus, if adults perceive cost as a barrier to a sustainable diet, it may indicate that adolescents would also, since they usually have less disposable income. Likewise, a study involving adolescents also found cost to be a barrier [45]. In addition, taste also reported barriers to consuming sustainable produce amongst adolescents [38, 39].

Concerning motivation, reflective motivation – beliefs about consequences, participants were confused, and a gap existed between their perceptions and the scientific literature. This same disconnect was also identified in adult populations [37]. A further barrier is that many felt uncertain and misled when they saw the size of dairy and animal-sourced proteins. Participants reported that their parents and schools advised them to consume larger portions of dairy and animal-sourced proteins to support bone growth and muscle development, respectively.

Nonetheless, the most significant facilitator promoting the adoption of a sustainable diet is its health benefits. Although concerns have been raised about whether a sustainable diet suits individuals with different dietary needs, most believe that adopting such a diet would benefit their health in the long term. Improving health as a primary motivation for adopting a sustainable diet has been recognised in adult cohorts [9, 13, 47–49]. Therefore, perhaps the focus should be on the health benefits of a sustainable diet rather than its environmental advantages to encourage its adoption, with positive environmental effects as an ancillary benefit.

However, pertaining to reflective motivation – social/professional role and identity and beliefs of capabilities, a key barrier identified by participants was that their current diet is so misaligned with a sustainable diet that they lack the confidence to make such changes. Likewise, Eustachio Colombo et al. [38] reported that although students valued the environmental impact of food, they would find it difficult to make the change. Similarly, the main obstacle concerns their cultural identity and their struggle to reduce it. Attachment to meat is a common challenge and was also recognised in many studies [9, 12, 22, 41, 49].

Fundamentally, regarding reflective motivation – intentions and automatic motivation – emotion, the vast majority were unwilling to adopt such a diet, with some expressing anger at the prospect of a transition. Unwillingness to adopt a sustainable diet has been reported in many studies on this topic, with Collins et al. [50] reporting that only half of the students included were willing to change their diet in order to reduce its ecological footprint. However, Collins et al. [50] also found that many students seemed ready to change to locally produced or organic food, with Derler et al. [51] reporting regional and seasonal foods to be important in the diets of adolescents. Likewise, Hielkema and Lund [52] noted that participants largely opposed reducing meat consumption. Culliford and Bradbury [48] reported low intentions to increase consumption of plant-based proteins, and Havermans et al. [36] found that adolescents showed little interest in adopting plant-based meals because they were perceived as having inferior taste. Furthermore, most participants closely resembled the findings reported by studies depicting that participants have high levels of resistance to adopting a sustainable diet [12, 22].

### Directions for future research, strengths, and limitations

Given the limited research on sustainable diets, various approaches can be explored to encourage the population to adopt sustainable eating habits. So far, the data on consumer barriers and facilitators to sustainable diets overwhelmingly expose how consumer knowledge and, in particular, their willingness to adopt a sustainable diet are low. Since this age cohort is primarily in education, schools can act as facilitators in this transition. Future research should explore modelling an intervention grounded in behavioural change theory. The BCW can provide the means of developing such an intervention or tool to address identified barriers to sustainable eating and make it a feasible choice for adolescents.

A strength of this study is that, to the researchers’ knowledge, it is the first to explore the barriers and facilitators to adopting a sustainable diet among adolescents in Ireland. It is also one of the few studies in Europe focusing on those under 18. Furthermore, although non-probability convenience sampling was used, participants in the selected cohort were randomly selected by the chosen gatekeeper. They were therefore unlikely to participate solely because they had an interest in the topic being explored.

Despite its strengths, this study has limitations that should be acknowledged. First, females constitute the majority of participants, as three of the seven focus groups consisted entirely of females. Three scoping reviews have also observed that participant samples in studies on sustainable diet research tend to be skewed towards women [5, 7, 16]. Additionally, although data were gathered from multiple sites, the small sample size typical of qualitative research means these findings may not be widely generalisable.

A further limitation of this study is that socioeconomic information was not collected from participants. Socioeconomic factors may influence the ability to adopt a sustainable diet and could therefore affect the observed associations. As a result, it was not possible to examine whether the observed findings varied according to socioeconomic background or to adjust for these factors in the analyses. Future research should consider including appropriate measures of socioeconomic status to better understand their potential influence on the outcomes of interest and to improve the generalisability and interpretation of the findings.

## Conclusions

Unless change occurs, the adoption of a sustainable diet is unlikely to happen soon, as barriers vastly outnumber facilitators. This study aimed to explore the barriers and facilitators to adopting a sustainable diet among adolescents in Ireland. The findings largely complement similar research in this area. Furthermore, the data found that psychological capability – knowledge and behavioural regulation was reported to act as both barriers and facilitators to the adoption of a sustainable diet, while physical capability – skill is a main barrier to sustainable diet adoption. Social opportunity – social influences, and physical opportunity – environmental context and resources were reported as barriers. Reflective motivation – beliefs about consequences was reported to contain both barriers and facilitators, while reflective motivation – social/professional role and identity, beliefs about capabilities, and intentions were reported as barriers towards sustainable diet adoption. Likewise, automatic motivation – emotion was a reported barrier.

Evidently, barriers (*n* = 33) were more frequent than facilitators (*n* = 8), and the vast majority admitted that transitioning to a sustainable diet is not something they are willing to pursue at present. The findings highlight the need for food literacy and indicate that behaviour change intervention frameworks, such as the BCW, are required to shift adolescents’ attitudes and behaviours by removing barriers and making sustainable options more convenient and accessible.

## Supporting information

S1 File Focus group schedule. (DOCX)

S2 File Accompanying quotes for the coding of the barriers and facilitators to the TDF of the COM-B model. (DOCX)
