# Supplementary material for: A qualitative study of the barriers and facilitators to adopting a sustainable diet among adolescents in Ireland
Source: PLoS One. 2026 Oct 27;21(10):e0361509. doi: 10.1371/journal.pone.0361509 (PMC13712759; doi:10.1371/journal.pone.0361509)
Supplement: Supplementary file 1 [file pone.0361509.s001.docx]

**Appendix A. Focus group schedule**

| **Questions** | **Prompts** | **TDF** |
| --- | --- | --- |
| *Introduction* | *Reinformed participants of their rights as outlined in their signed and returned assent/consent forms.*  *Audio recording began.* | |
| What do you know about the term ‘sustainable diet’? | What do you think are the features/characteristics of a ‘sustainable diet’  How do you think this diet compares to a healthy diet? | Knowledge  Beliefs about consequences |
| *Teaching of a sustainable diet* | *Using the Eat-Lancet sustainable diet plate by Willett et al. (2019) as a visual aid, the researcher explained to the participants what a sustainable diet looks like (see Figure 1). Considering the age of the participants, the researcher defined a sustainable diet as one that focuses on keeping both individuals and the planet healthy. It involves selecting foods that require fewer resources (such as water and land), generate less pollution, and do not contribute to climate change. A sustainable diet also focuses on eating more plants (like vegetables, fruits, beans, and whole grains) and less animal products (red meat and dairy in particular). A sustainable diet should also be accessible, affordable, and culturally acceptable. The researcher then gave examples of types of foods in each of the food groups.* | |
| What is your opinion of a sustainable diet? | What is your initial reaction to a sustainable diet? | Beliefs about consequences  Emotion |
| How do you think a sustainable diet could impact your health? | Not applicable | Beliefs about consequences |
| How do you think red meat production affects the environment? | Do you think these effects are mostly positive or negative or neutral? Explain | Knowledge  Beliefs about consequences |
| What are the factors that affect the foods you eat? | Do you set any personal goals regarding your dietary choices? | Environmental context and resources  Social influences  Goals |
| What are some challenges you think people **your age** may face if they want to eat a more sustainable diet? | Not applicable | Environmental context and resources  Beliefs about capabilities  Skills |
| How do you think your current diet/food choices might affect the environment? | How do your diet/food choices compare to the sustainable diet discussed? | Knowledge  Beliefs about consequences |
| What is the single biggest thing you could do to make your current food choices more sustainable? | Do you find it easy to remember or plan to include sustainable foods in your meals? What helps or hinders you? | Intentions  Behavioural regulation  Memory, attention, and decision processes |
| If you had to adopt a sustainable diet, how would you feel about doing so? | What would be your initial reaction if you had to adopt a sustainable diet?  Can you see eating sustainably as part of who you are, or as part of your identity? | Emotion  Beliefs about capabilities  Social/professional role and identity |
| Is it realistic for you to follow? Explain | How confident are you that you or people your age could successfully adopt a sustainable diet? | Beliefs about capabilities  Optimism |
| What would be the biggest challenge for you if you were to adopt a sustainable diet? | Not applicable | Beliefs about capabilities  Environmental context and resources |
| Are there rewards or positive outcomes that would encourage you to eat a sustainable diet? | Not applicable | Reinforcement |

**Visual aid provided to each participant to understand the concept and composition of a sustainable diet**

**Source(s):** The figure is informed by the EAT–Lancet Commission's Planetary Health Diet recommendations but is not identical to the original image and has been independently created for illustrative purposes only. Adapted conceptually from Willett et al. [1].
