# Supplementary material for: A qualitative study of the barriers and facilitators to adopting a sustainable diet among adolescents in Ireland
Source: PLoS One. 2026 Oct 27;21(10):e0361509. doi: 10.1371/journal.pone.0361509 (PMC13712759; doi:10.1371/journal.pone.0361509)
Supplement: Supplementary file 2 [file pone.0361509.s002.docx]

**Table 2.** **Accompanying quotes for the coding of the barriers and facilitators to the TDF of the COM-B model.**

| **COM-B Component** | **TDF Domain** | **Code** | **Quote(s)** | **Barrier (B) or Facilitator (F)** |
| --- | --- | --- | --- | --- |
| Capability (Psychological) | Knowledge | Unfamiliarity with plant sources of protein | *I did not know that beans had protein in them, I won’t lie.”*  FG7, P3  *“I don’t even know what tofu is.”*  FG2, P2 | B |
|  |  | Unawareness of the characteristics of a sustainable diet | *“Is it like eating healthy every day, not just one day?”*  FG6. P5 | B |
|  |  | Unsure of how red meat production affects the environment | *“I don't know too much about it, but I do know that there's a whole thing going on, the gases that cows produce, like it's damaging the environment. So, I think that would be like an environmental reason why people would try to cut down on their consumption of beef. But I don't know so much about it.”*  FG3, P6 | B |
|  |  | Awareness that a sustainable diet keeps resources for future generations | *“A diet that can last over the years, like you're not wasting resources and stuff.”*  FG6, P3 | F |
|  |  | Understanding that a sustainable diet does not damage the environment | *“A diet you can eat that can keep you healthy, as well as not negatively impact the environment.”*  FG1, P6 | F |
|  | Behavioural Regulation | Current diet does not align with a sustainable diet | *“I would say dairy would probably be a good lot, a big chunk of it now, I would say, and probably meat.”*  FG6, P7  *“I'd never eat that many oils.”*  FG3, P6  *“The plant-source protein, I have none of that.”*  FG6, P3  *“I definitely don’t eat that many vegetables.”*  FG2, P2  *“My animal protein would probably be like a quarter of a plate.”*  FG3, P4  *“Yeah, not to be very stereotypically Irish, but I have potatoes maybe five or six times a week with dinner. So, I think, like, the starchy vegetables, there'd be a lot more for me.”*  FG2, P4  *“I feel like I'd have a lot more sugar because of how much coffee I drink.”*  FG2, P5 | B |
|  |  | Acknowledgement of the changes required to make their diet more sustainable | *“More plant-sourced proteins as well. I could throw a few beans into something.”*  FG6, P5  *“I just think that I need to reduce the animal-source protein, and I thought it would be a way bigger.”*  FG5, P3  *“I feel like if I put more fruit and veg with every meal, everything else will kind of get smaller.”*  FG6, P5  *“Plant-sourced protein and unsaturated plant oils, I should probably make them much bigger.”*  FG5, P8  *“I think I probably need to eat more whole grains as well.”*  FG2, P1  *“I should cut down on dairy in my diet.”*  FG5, P2  *“Probably reduce whole grains.”*  FG1, P9  *“Decrease starchy vegetables, like eating less potatoes and stuff.”*  FG4, P3  *“I would have to cut down on added sugars, as I love chocolate.”*  FG1, P1 | F |
| Capability (Physical) | Physical Skills | Lack of practical skills to prepare sustainable meals | *“A lot of parents only eat animal sources of protein; let’s say they don't know how to cook tofu, and then they can't use it”*  FG4, P6 | B |
| Opportunity (Social) | Social Influences | Parents buying and preparing food | *“You can only really eat what your parents are buying and making for you, so if you want to eat like this [sustainably], and your parents are like, I am making this dinner, and you can either eat it or not eat it.”*  FG4, P2 | B |
|  |  | Peer pressure shaping food choices | *“So many of my friends go to the canteen, and I tell them to come along. You just have to get something. Everyone else is sitting there, and it just smells so nice.”*  FG2, P3 | B |
|  |  | Influence of social media on food choice | *“If you're seeing more unhealthy things, you might want to eat less healthy. But if you see more people like influencers, eating healthy might influence you to eat healthier food.”*  FG3, P6 | B/F |
|  |  | Socially acceptable | *“I think if you're surrounded by the right friend group, nobody would judge for what you decide to eat, you know.”*  FG3, P2 | F |
|  |  | Socially unacceptable | *“You would get slagged if you've got a big bundle of salad, you would get called a ‘rabbit’ or something.”*  FG5, P8 | B |
|  |  | Social norm clashes | *“You need meat to grow, especially when you're young, so my mam always says.”*  FG2, P5  *“Yeah, and like a lot of people say, we should drink milk and stuff like for calcium, you can get nutrition from dairy.”*  FG2, P1 | B |
| Opportunity (Physical) | Environmental Context & Resources | Lack of access to sustainable foods | *“This is something I experience, like when I go to the supermarket here [Offaly], there are barely any options, whereas my sister is going to college in Dublin, and if I ever visit her, there are way more options.”*  FG5, P1 | B |
|  |  | The school canteen has limited options | *“There is not really a very healthy option [at school], as they don't do salads. They don't like things with vegetables in them; it's just animal protein they use.”*  FG1, P4 | B |
|  |  | Convenience as a factor affecting food choices | *“Convenience like. Say if you're out in town after school or something, a takeaway is handy instead of going into the shop and buying vegetables. You have to cut them up and make stuff out of them.”*  FG6, P6 | B |
|  |  | Inconvenience of a sustainable diet | *“A sustainable diet is less convenient because of its preparation; you have to peel most vegetables, wait for them to boil, and chop them. Whereas with meat, you can just put it in the oven or the pan.”*  FG3, P2 | B |
|  |  | Cost shaping food choices | *“If it is the end of the week and you don't have as much money left or something, you might just like to get something cheaper, but it might not be as good for you.”*  FG2, P4 | B/F |
|  |  | Stage of life clashing with a sustainable diet | *“I think it'd be more realistic if I were older. If I like, live by myself or something. Or if I were making dinners for myself and I was on like, my schedule, it would be a lot easier.”*  FG2, P3 | B |
| Motivation (Reflective) | Beliefs about Consequences | Viewing a sustainable diet as positive for human health | *“It'd probably be better for your skin, for your teeth, for your hair, for your eye health, and probably be better for your gut too … it's also better for maintaining your weight, for losing weight.”*  FG1, P8 | F |
|  |  | The perception that a sustainable diet would not be satiating | *“I think you would be a lot hungrier because I suppose the majority of my energy comes from starchy vegetables and stuff like that, and whole grains. But I just feel like I'd be so hungry if half my plate were vegetables. I’d feel like I'd have no energy.”*  FG3, P1 | B |
|  |  | Perceiving a sustainable diet as expensive | *“Just how expensive vegetables and fruit would be.”*  FG5, P4 | B |
|  |  | The amount of dairy/meat/starch is too small | *“I just always thought that people were encouraged to eat meat because it's still good for you and has so much protein in it.”*  FG2, P3 | B |
|  |  | A lot of ‘harmful’ oils | *“I was surprised by the unsaturated plant oils. I thought they would be way smaller because oil is bad for you, so I presumed it would be a good bit smaller than that.”*  FG6, P7 | B |
|  |  | Concerns surrounding the generalisability of a sustainable diet | *“Like children are obviously growing very fast, I don't think that'll be enough protein for a child, or like a pregnant woman who would need an awful lot more protein.”*  FG3, P2  *“It doesn't really take into account things that people might already have. Like, if someone has something like heart disease or something like that, they probably shouldn't be eating as much of the oils and things like that.”*  FG3, P5 | B |
|  |  | The healthiness of food determines food choices | *“I think the health of it as well, because being in home economics, and even hearing on the radio and stuff of how you should be having more of say, fibre and less sugar and if you see something in the shop that says more fibre, less sugar or something like that on the packet, you'd be more likely to buy that.”*  FG4, P6 | F |
|  |  | Sustainable diet adoption is adversely impacting the Irish economy | *“We transport a lot of meat, which means we get a lot of money, so part of our income comes from meat, so it has its positives as well. In Ireland, it's a large proportion of people's income.”*  FG4, P5 | B |
|  |  | Belief that red meat production has a positive environmental impact | *“I think there would be an overpopulation of cows if they were not used for meat.”*  FG3, P6  *“Cows aren’t really a cause of climate change.”*  FG7, P1 | B |
|  |  | Belief that red meat production has a negative environmental impact | *“Land use for all the fields and farms, like you have to cut down trees which take in carbon dioxide, and all the machinery used as well, there are a lot of emissions from that.”*  FG5, P8 | F |
|  |  | Belief that red meat production has a neutral environmental impact | *“They probably balance each other out, kind of, because they both can be negative. Ireland is known for beef and stuff. And then if you're bringing, like, avocados, from God knows whatever, you know, the travel costs the environment a lot to bring them over.”*  FG6, P7 | B |
|  |  | Transportation of vegetables is worse than that of local meat | *“If you grow fruit and veg, you can’t grow a lot of them in Ireland, and if you have to get them from other countries, that would be worse for flying them over or shipping, and that would be worse for the environment, I think.”*  *FG6, P1* | B |
|  | Social/Professional Role & Identity | Cultural influence on diet | *“I think it probably would be [easy to adopt] if you’ve grown up with it, but because we have not grown up with it, it is not our comfort food. It's not like the cultural food that we've been eating.”*  FG1, P3 | B |
|  |  | Attachment to meat and dairy | *“That could be very difficult because people are so used to eating what they’re eating, like especially animal-sourced protein, like meals revolve around that, like pick a meat out fridge and then go ok, I'm going to make something with this meat and then revolve the rest of my dinner around that.”*  FG4, P1 | B |
|  |  | Attachment to starch | *“Yeah, not to be very stereotypically Irish, but I have potatoes maybe five or six times a week with dinner. So, I think, like, the starchy vegetables, there'd be a lot more for me”.*  FG2, P2 | B |
|  | Beliefs of Capabilities | Struggle to reduce meat and dairy | *“I think the majority of people our age will at least eat meat with like two of their meals, like I could eat meat with all three of my meals. I could eat meat with my breakfast, my dinner, and my supper, so it'd be very difficult to cut down on that when it's a big focus on the majority of my meals like.”*  FG3, P5 | B |
|  |  | Lack of confidence to change diet | *“It sounds silly, but nervousness, like if you're changing up your entire diet and menu plan, I think it can be a bit daunting because if you're trying to do things you haven't tried before it could be a waste of money if you buy certain foods and you don't like them.”*  FG4, P6 | B |
|  |  | Believing change is possible | *“I feel like that wouldn't be terribly hard, though, because you can use tofu as a replacement for a lot of different types of meat, and then lentils. You can't replace them the same way as you could replace chicken or something; you can add it into sauces.”*  FG1, P9 | F |
|  |  | Diet is too dissimilar to make a change | *“I'm half Filipino, so are our plates like especially like with male Filipinos, it'll be at least half rice and then just animal protein. Not many vegetables. There might be some vegetables, but I'd say it's a majority of rice and meat.”*  FG1, P1 | B |
|  | Intentions | Unwillingness to adopt a sustainable diet | *“Like, obviously, there's always a select few that are different. But I think the majority have no intention of following that [sustainable diet].”*  FG3, P4 | B |
|  |  | Change would take time | *“I would have to change slowly into it because it's just not sustainable if I were to hop straight into the diet; it's just not what I'm used to.”*  FG7, P2 | B/F |
| Motivation (Automatic) | Emotion | Dislike of vegetables | *“I am shocked there is that much veg. Like I knew there'd be a lot, but I didn't think they'd be half your diet.”*  FG2, P4 | B |
|  |  | Negative reaction to following a sustainable diet | *“I’d hate it if I had to eat like that”*  FG2, P5 | B |
|  |  | Shocked at the number of plant-sourced proteins in a sustainable diet | *“Definitely increasing plant-sourced protein because I'm not a fan of things like all tofu.”*  FG3, P2 | B |

**Source(s):** Authors own work
